# How and why are video consultations used in urgent primary care settings in the UK? A focus group study

**DOI:** 10.3399/BJGPO.2023.0025

**Published:** 2023-06-14

**Authors:** Rebecca Elizabeth Payne, Aileen Clarke

**Affiliations:** 1 Nuffield Department of Primary Care, University of Oxford, Oxford, UK; 2 Public Health and Applied Health Research, Warwick Medical School, University of Warwick, Coventry, UK

**Keywords:** telemedicine, remote consultation, urgent care, general practice

## Abstract

**Background:**

Video consulting was widely rolled out across general practice at the start of the COVID-19 pandemic. In the in-hours setting there has been a marked shift away from using the technology, but many urgent care clinicians continue to use video consulting. Little is known about the reasons behind this discrepancy.

**Aim:**

To understand how and why video is used in urgent care settings.

**Design & setting:**

Focus groups were held via Microsoft Teams with 11 GPs working in in- and out-of-hours settings across the UK.

**Method:**

GPs were recruited through a convienience sampling strategy. Meetings were recorded, auto-transcribed, and checked for accuracy. A thematic analysis was performed.

**Results:**

Urgent care GPs used video as an adjunct to the telephone in the initial assessment of patients and felt it helped direct patients to the right service first time. They were confident using video for a broad range of presenting conditions. They felt it created additional trust and rapport with patients and was useful for bringing third parties into the consultation. They felt that it allowed them to maximise resources and use shielded colleagues effectively. They emphasised the importance of one-to-one training and this was seen as vital for effective implementation within an organisation.

**Conclusion:**

Video consulting is useful in the urgent care setting as an adjunct to telephone consulting. It is particularly helpful in the initial triage of patients. One-to-one training is needed for effective implementation.

## How this fits in

Video consulting was introduced widely to UK general practice and urgent care settings at the start of the COVID-19 pandemic. In-hours general practice usage has declined considerably. This study highlights how urgent care GPs continue to use video consulting, finding it particularly helpful as an adjunct to the telephone in the initial assessment of patients to create rapport, reduce complaints, and to maximise resources.

## Introduction

Urgent care services, such as NHS 111 or GP out of hours, are characterised by a primary purpose of addressing urgent medical issues. Their focus is on getting the patient the right care in an appropriate timescale to address immediate medical needs such as respiratory tract infections, paediatric fever, and abdominal pain. Doctors in such services need to quickly gain rapport and create trust so that the patient can feel confident to adhere to their recommendations.

UK urgent primary care services cover a large geographical footprint. To access the services, a patient calls 111 and speaks to a non-clinical call-handler who uses algorithms, such as NHS pathways,^1^ to direct the caller to the appropriate service for their needs. There are multiple possible outcomes for a call including advice from a call-handler or clinician, diversion to pharmacy, or a face-to-face appointment.^2^ Video consultation is commonly implemented as an adjunct to an initial telephone consultation^3^ and a patient often proceeds to a face-to-face appointment.

Video consulting is regularly used in the urgent care setting. This is in marked contrast to in-hours general practice. A recent article identified a range of reasons why GPs have stopped using the technology. These included the lack of additional advantage over a telephone call, technological limitations, and clinician preferences for face-to-face care.^3^

This study examined why video is more popular in the urgent care setting through interviewing GPs from both sectors in a focus group setting.

## Method

### Design, setting, and sample

A qualitative study using focus groups of UK-based GPs was conducted. All GPs met the inclusion criteria of having used video consultation in primary care since the start of the COVID-19 pandemic in March 2020 and worked in a range of settings across all four nations of the UK. Some GPs continued to use video consulting regularly, others had moved away from regular use of the technology. Both in-hours and urgent care GPs (out of hours and NHS 111) participated in focus groups held online via Microsoft Teams.

A convenience sampling strategy was used for recruitment. Adverts were placed on social media sites such as GP Facebook groups and Twitter.

Three focus group sessions were planned. Sixteen GPs expressed interest, and 11 participated in the three focus groups. Five of the participating GPs worked in urgent care.

Potential study participants were contacted via email and sent a participant information sheet and a consent form. Participants gave informed consent to participate under Chatham House Rule.

### Characteristics of participants

Characteristics of participating GPs are listed in Table 1. GPs were from a wide range of career stages and UK nations, with no demographic differences seen between urgent care and in-hours GPs.

**Table 1.** Characteristics of participating GPs (*N* = 11)

**Characteristic**	***n***
**Sex**	
Male	3
Female	8
**Years qualified as a GP**	
0–5	2
>5–10	2
>10–20	5
>20	2
**Country of practice**	
England	8
Northern Ireland	1
Scotland	1
Wales	1
**Type of work where video consulting was used^a^**	
Urgent care (NHS 111, out-of-hours urgent treatment centres, and COVID hub)	5
In-hours practice	9
**Setting**	
Rural	2
Urban	1
Mixed	8

^a^Some GPs worked in more than one setting.

### Focus groups

Three focus groups were held via Microsoft Teams in December 2021. Groups contained between three and five GPs. An interview guide was used to ensure consistency and was formulated according to the Leung and Savithiri (2009) framework detailed in Table 2.^4^ The groups were chaired by the lead author, a GP experienced in video consulting. The sessions were lively, with conflicting opinions expressed between the GPs and detailed discussion of the issues. Saturation had been reached by the end of the third focus group.

**Table 2.** Interview guide based on the Leung and Savithri (2009) framework^4^

Category of question	Question used
Opening question	Can you introduce yourself to the group please?
Introductory question	What’s been your experience of video consulting?
Key questions	What [patient] presentations do you think are suitable for video consultations?Any presentations definitely not suitable for video consulting?Have you had any significant events in relation to video consulting?
Concluding questions	Anything else you feel we should be aware of?

Meetings were recorded and auto-transcribed via Microsoft Teams, then manually checked for accuracy. Verification was performed through a senior qualitative researcher reviewing a call recording of one of the three focus groups.

An inductive approach was taken to analysis, using grounded theory. The call recordings were listened to repeatedly in order to immerse the researcher in the data and allow nuances in tone and wording to be detected, and the voice of the GPs heard and understood. This provided an initial impression of themes that were written down. Following this immersion, the Microsoft Teams auto-transcripts were downloaded, exported to Microsoft Word, and printed. These were then manually coded using colouring pencils and themes identified and compared with the initial set of themes identified through the listening exercise. Final verification of interpretation was performed through listening back to the recordings in order to ensure all themes had been captured and that the auto-transcription was correct.

The lead author conducted the analysis, concentrating on the results in relation to urgent care settings.

## Results

A number of themes emerged from the focus groups. One of the most marked was the very different attitudes towards the use of video consulting by the five doctors using video in urgent care settings compared with the six working solely in traditional GP settings. Urgent care GPs were more enthusiastic about the technology and used it frequently. Themes emerging from an analysis of the comments from the urgent care GPs are shown in Box 1 and expanded on below.

Box 1 **Themes from urgent care doctors**

- Video consultation was useful for the initial assessment of patients.
- Video allowed an additional level of rapport and trust between clinician and patient, and reduced complaints.
- Video consulting had value in a wide range of conditions including in identifying safeguarding issues.
- Video was useful for bringing others into the consultation — either to assist with physical examination or to get specialist advice for patients.
- Video helped maximise resources and used shielded colleagues effectively.
- Training was vital in order to overcome staff anxieties and get them confident using the technology.

### Lack of enthusiasm from in-hours GPs

All of the in-hours GPs were considerably less enthusiastic than their urgent care colleagues. They felt that video consultation had value for a much more limited range of conditions, with one GP stating it was *‘Not the revolution I was expecting’* (Female GP, in-hours, late career)*.* They felt that call set-up took too long, taking up time allocated for the appointment. Video consulting did not fit well within practice processes, with it often being quicker to ask a patient to attend the surgery.

*‘Video is a bit of a faff to organise, especially if you’ve got an elderly patient, I spend so much time getting it set up.’* (Female GP, in-hours, mid-career)

Even GPs who used video consulting regularly in the out-of-hours setting did not use it in their surgeries.

### Adjunct to initial assessment of patients

All urgent care GPs used video consultation during the initial assessment of patients. GPs started a consultation by phone and then switched to video if they felt it would give them relevant additional information. They found it helpful in the following circumstances:

First, to safely prioritise patients, identifying those who were seriously unwell and escalating their care. Several of the doctors mentioned how their 'sixth sense' came into operation on video calls:

*'… the video gave me the gut reaction of going: “That child doesn't look well … ” I've looked at this child and their respiratory rate* [was] *up and that could have been very different had I just not been going with the sort of light “Spidey-sense”* [which told me] *…. this isn't right … '* (Female GP, urgent care, mid-career)

In contrast, in-hours GPs commented that if they were worried about a patient they would ask them to come in for an appointment:

*'If I’m worried about someone I always want to see them face to face.'* (Female GP, in-hours, mid-career)

In-hours GPs felt that video offered little additional benefit over a telephone call:

*'For that rapid “Do you need to go to A and E, do you need an ambulance?”, telephone is all you need, if I’m worried I’ll always bring them in.'* (Male GP, in-hours, mid-career)

Second, to provide justification for asking the patient to attend a face-to-face assessment. This was particularly so when the patient would have to travel long distances, common in rural areas during the out-of-hours period:

*'They were asking us to make parents do a 60- or 70-mile round trip, and video was really helpful in helping you decide if someone needs to be seen because you pick up “actually they look miserable, they need their vital signs done”*.*'* (Male GP, urgent care, late career)

*'If you’ve eyeballed them and they’re coming because you’ve seen them and you feel they need further assessment … it helps sell it to them.'* (Male GP, urgent care, late career)

In contrast, in-hours GPs, who covered much smaller geographical areas, found it more efficient to ask the patient to attend the surgery:

*'In a way just bringing a patient who’s 5 minutes away or right round the corner down to the practice is just quicker than trying to talk them through logging onto a system*.*'* (Female GP, in-hours, mid-career)

Third, to support decision making about which patients could safely be managed without a face-to-face appointment:

*'… When you get the video turned on, as a clinician you see a child who’s not in distress or struggling, and it just saved so many unnecessary contacts*.*'* (Female GP, urgent care, mid-career)

*'I find some people can sound really awful on the phone, but when you look at them on the video you can see instantly … they're actually all right and vice versa*.*'* (Female GP, in-hours and urgent care, mid-career)

In-hours GPs were much less confident treating patients without seeing them face to face compared with those in urgent care:

*'I mean, for me, anything that I need to listen to, you know so heart sounds for example, you know … And yes, you can get away to a degree with assessing how short of breath someone is, but you can't listen to their chest.'* (Female GP, in-hours, late career)

Fourth, for directing patients to appropriate services; GPs felt that being able to 'downgrade' a patient from an emergency department (ED) to a minor injury unit offered benefits both to the patient and to the wider system.

*'… Things like burns, assessing the amount of area they cover, how deep they are … '* (Female GP, urgent care, mid-career)

*'Often people will ring in with injuries. “I've had a fall”… and it'll be “is this broken? Is this not?” ... Often the video call helps filter what the appropriate setting is. Yeah, it might be broken, but a minor injuries* [department] *can cope with it.'* (Female GP, urgent care, mid-career)

In-hours GPs had different decisions to make; the question for them was generally not whether the patient was suitable for their services but whether the patient needed to come in to the surgery for further assessment. They therefore found less benefit in the additional information provided by the video over telephone, preferring to use photos:

*'I tend to find it quicker to do the photos thing, so I just send them a link and then go and deal with something else … rather than let’s get the video up, it would just take ages so we just don't have time ... '* (Female GP, in-hours, mid-career)

### Creating rapport

Many of the GPs felt they gained a deeper level of personal connection with patients over video compared with telephone and that this assisted with building rapport:

*'It was literally just like being in a in a face-to-face consultation. It felt a lot more personal. It felt a lot easier to talk to them because they could see me. I can see them … I managed to actually just gel with them and get that rapport quite quickly.'* (Male GP, urgent care, early career)

The GP medical director of a large urgent care service reported a much lower complaint rate relating to video consultations than telephone encounters, this was attributed to a greater sense of human connection on a video rather than a telephone call:

*'Often you don't know those patients and they don't know you. And I do think when you add video there’s an extra connection with that individual*.*'* (Female GP medical director, urgent care, mid-career)

In-hours GPs often already knew the patients they were treating, and did not mention the need to use video in order to increase rapport.

### Dealing with a wide range of conditions

The urgent care GPs felt video consultation was suitable for a wide range of conditions:

*'You can just say “pull up their T-shirt, show me their ribs”.'* (Female GP, urgent care, mid-career)

*'If I’m not really sure if it’s blanching* [checking a rash for signs of meningitis]*, you can clear that up quite easily over a video if you’ve got a good connection.'* (Female GP, urgent care, mid-career)

*'You can do the shoulder examination for frozen shoulder quite easily on video a lot of the time, getting them to move their shoulder.'* (Male GP, urgent care, early career)

The only area urgent care GPs reported they would never use video for was for problems viewing the breasts or genitalia. This was owing to legal concerns around images:

*'We certainly have a rule of no intimate images, no. So things like anything that would involve genital problems or breast problems would be an absolute veto*.*'* (Female GP, urgent care, mid-career)

Urgent care GPs particularly valued video consultation in helping identify incidental safeguarding issues:

*'We've incidentally picked up some safeguarding issues, so we've had situations where we've ended up sort of doing referrals, either for families that clearly need a bit of extra support, or where we've had concerns*.*'* (Female GP, urgent care, mid-career)

Other conditions the urgent care GPs felt particularly benefitted from video consulting were COVID-19 cases and paediatric disorders, such as fever, where video consultation provided an extra degree of reassurance to both clinicians and parents.

There was consensus among both groups of GPs that video consultation was less appropriate where there were multiple comorbidities, particularly in older people, or vague or undefined symptoms:

*'I would find our complex comorbid elderly patients who struggle to hear… I just find video doesn't work with them; it just gets so complicated … it’s a bit like having a family zoom. You know, it doesn't really work because everyone talks over the top of each other and no one really communicates properly. And so those sorts of things I found are just better face to face … '* (Female GP, in-hours, late career)

Other areas the GPs generally felt were less suitable for video consultations were tonsillitis, ears, cardiac, respiratory issues (other than COVID-19), and abdominal pain. In general, the urgent care GPs were more confident than the in-hours GPs using video consultation for a wider range of issues.

### Bringing third parties into the consultation

Two of the urgent care doctors mentioned that 'facilitated video consultation', where a relative or carer performed an enabling function, were particularly helpful as they gave clearer images and minimised technological disruption:

*'I had one kid where I used the mother as a sort of "avatar" effectively ... The mother was sensible, able to follow instructions. … this kid was really quite sore and was admitted and it was appendicitis … I mean, it’s like that sort of Holy Grail of all the things coming together, the history and a sensible parent and a child that was willing … '* (Female GP, urgent care, mid-career)

Urgent care GPs also valued using video to add an on-call specialist into a consultation:

*'It was 10 miles to the nearest hospital, 20 miles to the nearest one with a surgical team and 40 miles from the vascular unit. We had a three way consultation with the vascular surgical reg and in the end we decided he … could be seen locally and in the end the diagnosis was probable DVT* [deep vein thrombosis]*,* [his limb] *was cold because of venous congestion.'* (Male GP, urgent care, end career)

### Maximising resources

Video consulting allowed maximisation of scarce medical resources, particularly in the out-of-hours period, in under-doctored areas of the country, in rural areas, and at times of system pressure. Many of the doctors were uncomfortable with this, feeling that video technology was used to provide substandard care:

*'… they would use a lot more video rather than patients drive, you know, 30 miles and 60-mile round trip in order to get a normal appointment … That again is compromised. That’s using the video to get away with not actually having sufficient clinicians to provide a service in in a rural area.'* (Female GP, in-hours, late career)

It allowed staff to be utilised more effectively; for example, shielded doctors during the pandemic, as well as those doctors who would ideally prefer to conduct a face-to-face consultation were reported as being happier to work remotely if video was provided:

*'… we found it completely invaluable, especially where we had a lot of workforce that might have been either at higher risk of COVID and* [who] *were very concerned about still seeing patients face to face. This was a bit of a lifesaver … '* (Female GP, urgent care, mid-career)

The resource that in-hours GPs focused on most was GP time. They were concerned that video calls took up appointment time, but gave little additional value over a telephone call. They commented on the awkwardness of trying to set up video consultations with patients and were alert to the challenges of both affluence and poverty as well as age. A rural GP reported patients with large houses had Wi-Fi blackspots, while those on lower incomes often had poor resolution to their phone cameras or limited data.

*'… our very affluent patients. Their houses are so big that they have rubbish Wi-Fi and so you know you're kind of screwed either way.'* (Male GP, in-hours, mid-career)

*'We had elderly folks who were fantastic because they had great experience talking to their grandchildren and WhatsApp or whatever, and we'd others who just couldn't fathom it at all*.*'* (Female GP, in-hours, mid-career)

### Training and infrastructure vital

It was recognised that doctors often needed support to familiarise themselves with the technology before they felt confident using video, and that systems needed to be user-friendly. Written instructions were inadequate to get doctors to do video calls; one-to-one training time was needed:

*'… especially for some of my colleagues who perhaps felt less comfortable with the IT we did notice we had to do a bit of sitting down with them and literally talking them through, actually doing a dummy call with them and saying, “look … it’s really straightforward. This is what you do. This is how it works” so that they felt more comfortable, more confident with it … just sending out written instructions didn’t always work.'* (Female GP, urgent care, mid-career)

In contrast to the in-hours GP setting, urgent care services had invested in the technology to make video consulting easy and integrated video into workflows. GPs who were enthusiastic about video consulting in urgent care reported they didn’t use it in-hours:

*'So from a practice point of view, I've literally used it five times. I still don't have dual screens. Or you know, the headsets that would work so that patients could hear what you were saying and stuff.'* (Female GP, urgent care, mid-career)

## Discussion

### Summary

This study of GPs has explained why urgent care GPs find video consulting so helpful, compared with their in-hours colleagues. Their focus on getting the patient to the right service on the right timescale meant additional information gained via video at initial triage helped to identify and prioritise patients needing urgent care. Video supported decision making about which patients could be safely managed remotely, which provided extra reassurance to clinicians and patients, and where travel was needed, it allowed the patient to attend the most appropriate service for their needs. GPs believed this reduced system pressure through diverting patients from EDs to other clinical settings.

Video consultation provided extra justification when asking a patient to travel long distances for further care. Video consultation allowed resources to be maximised, using home-working staff and providing care to under-doctored regions. The compromises and trade-offs of this approach were recognised by doctors.

Video helped GPs to generate rapport with patients, which fed through to a lower rate of complaints.

Urgent care GPs were happy to use video consultation for a wide range of conditions. They valued its ability to identify incidental safeguarding concerns. They found it particularly helpful when a carer or relative was able to help facilitate examination; for example, palpating a child’s abdomen. Video consultation allowed GPs to get rapid specialist advice for patients still at home, through adding a third party into a call.

Training and infrastructure was recognised as vital, and a large part of why video consulting was effective in the urgent care setting when compared with GP practices.

There are several factors inherent to the urgent care environment that make video consulting effective. The long distances that patients may have to travel and the limited capacity within out-of-hours centres and EDs mean that spending more time gaining additional information remotely via video is beneficial if it saves a face-to-face appointment, or diverts the patient to a lower-acuity setting.

Patients consulting urgent care services are likely to be different to in-hours patients, in that they all have or perceive themselves to have an urgent medical need. In this patient cohort, video consultation can provide important additional information to identify seriously unwell patients or raise safeguarding concerns.

In urgent care, because doctors are unknown to the patients, any additional rapport gained is significant as the consultation represents the one and only chance to establish trust. The rapid establishment of this therapeutic relationship by video allows the patient to feel reassured in a way they may not following a telephone consultation.

### Strengths and limitations

The study sampled GPs working across the UK in a range of different primary care roles. The GPs were from a wide range of career stages. Mixed focus groups of in-hours and urgent care GPs allowed different attitudes towards video consulting in the two groups to be explored in-depth.

Although saturation was reached, the convienience sampling strategy and small numbers risk not representing the full range of GP views on video consulting. Advertising on social media meant some GPs were not reached and it may have led to selection bias with more technologically confident GPs participating in the study. Lack of remuneration deterred some GPs from taking part, although payment might have influenced the findings, especially if GPs felt there was an incentive to be positive about the technology.

The call recording for one of the focus groups was reviewed by a senior qualitative researcher; however, the transcripts were analysed by a single reviewer. Best practice would be for transcripts to be reviewed and coded by two different reviewers working separately and then together.

The focus groups were chaired by a GP working primarily in urgent care, who regularly uses video consulting in her own clinical practice. This may have risked bias to the discussion and analysis. Attempts were made to minimise this through the GP chairing rather than contributing to the discussions, and through the involvement of colleagues in verification and analysis.

A final limitation is that the study only looked at GP views. The views of patients and other primary care staff are vital to include as services are developed, but this was beyond the scope of this study. The study also did not examine patient outcomes. More quantitative study designs are needed to understand the impact of video consulting on individual patient outcomes and wider system performance.

### Comparison with existing literature

A key part of urgent care services is recognising and treating acute illness. Early on in the pandemic there were concerns that acute illness was less suitable for video consulting,^5,6^ but the clinicians in this study found it particularly useful for acute illness. This is in line with other findings in pre-hospital care, where video consulting has been found to be helpful in assessing patients with stroke,^7,8^ burns and trauma,^8^ and mental health.^9^

Another fundamental role of the telephone-based NHS 111 service is to direct patients to suitable further care on an appropriate timescale. Other studies have found video consulting is helpful for ensuring that those who need further care are directed to the right setting.^10,11^ This study found that being able to redirect patients to a lower-acuity care setting by gaining additional information through a video call was valued by urgent care clinicians. The system benefits are unquantified but important in the context of ED pressures, as accurate ascertainment of severity allows only appropriate patients to be directed to these services. Previous studies have demonstrated that dispositions can be downgraded with the additional information provided by video,^10,12^ including where a patient has called 999.^13^

In urgent care services, where clinicians are usually unfamiliar with patients, gaining a quick therapeutic rapport is vital. This study found an increase in rapport when video was used, similar to findings from other studies.^14,15^

The findings on video helping to identify safeguarding issues are in contrast to other research that has found remote consulting a barrier to picking up safeguarding concerns, although much of this body of work focuses on telephone rather than video consultations.^16^

The training issues highlighted by the doctors are important. Other studies have found that peer training is useful to introduce non-early adopter GPs to video consulting,^17^ and that there is a correlation between a desire for more training and the non-adoption of video consulting.^18^

The infrastructure findings strongly echo other studies on this subject.^3^ One of the reasons that urgent care services are able to use video effectively is that it has been integrated into their workflows, and services have invested in dual screens and headphones.

Overall, this study adds further information about the presenting complaints where urgent care GPs feel video adds extra value, as well as about its use in conserving workforce resource and mobilising parts of the workforce that would otherwise be unavailable for patient-facing duties. It demonstrates how video can be used to bring third parties into a consultation, including to provide additional information by performing a limited examination under instruction.

### Implications for research and practice

Video consulting is a useful adjunct to the telephone in the initial assessment of a wide range of conditions in NHS 111 and other urgent care services. By enhancing the accuracy of initial triage it allows appropriate management of patients, potentially reducing pressure on ED and ambulance services. In the context of UK emergency sector pressures, it should be more widely adopted within the NHS 111 setting, particularly at times of system pressure. Box 2 contains recommendations for clinicians, urgent care providers, and for national bodies. These include using video to justify asking a patient to travel, the need for infrastructure to be addressed through headphones and dual screens, and the recommendation to adopt video consulting in all NHS 111 settings.

Much research has focused on video consulting in the in-hours sector; however, given it appears to show more benefits for both patient and system in the urgent care setting, focus should be placed on a shift into urgent care, with potential for greater funding. Areas for further research should include patient outcomes and re-presentation rates following a video consultation, as well as a deep-dive into which presentations are most suitable for video consultation. The cost-effectiveness of video consultation and it’s impacts on equity should also be explored.

Box 2 **Recommendations for practice**

**For urgent care clinicians**

- Adding in video following initial telephone assessment gives extra information that can assist with prioritisation and disposition.
- If you are going to ask a patient to travel, a video consultation can help justify that decision.
- When a contact is completed without a face-to-face consultion, both clinician and patient can feel more confident that the right outcome has been reached if video has been used.
- Video can be used to bring in third parties, such as a relative to assist with examination or a specialist at another site.
- Listen to your gut instinct when assessing a patient via video.

**For in-hours GPs**

- Video consulting may have particular benefits when assessing acutely unwell patients.
- Consider using video consultations to maximise practice resources, for example, by using clinicians working remotely.
- Infrastructure, such as dual screens and head sets, is needed to enable video consulting to work effectively.

**For urgent care providers**

- Invest in the kit to make video consulting easy, for example, dual screens and headsets.
- Provide one-to-one training for any clinicians reluctant to use the technology.
- Using video may reduce complaints and improve your ED and ambulance dispositions by allowing patients to be safely directed to a lower-acuity setting.

**For future research**

- Patient outcomes and re-presentation rates following video consultation.
- Which presentations to urgent care are suitable for video consultation?
- Cost-effectiveness of video consultation.
- How does video consultation affect equity and inequalities?

**For national bodies**

- Royal College of General Practitioners: consider providing bespoke training in video consultation for urgent care settings.
- NHS: adopting video consulting in 111 can divert more patients down lower-acuity pathways and should be promoted and funded.
